# Transoral fundoplication offers durable symptom control for chronic GERD: 3-year report from the TEMPO randomized trial with a crossover arm

**DOI:** 10.1007/s00464-016-5252-8

**Published:** 2016-09-21

**Authors:** Karim S. Trad, Mark A. Fox, Gilbert Simoni, Ahmad B. Shughoury, Peter G. Mavrelis, Mamoon Raza, Jeffrey A. Heise, William E. Barnes

**Affiliations:** 1 The George Washington University School of Medicine and Health Sciences, Washington, DC USA; 2 Crossville Medical Group, Crossville, TN USA; 3 Cumberland Medical Center, Crossville, TN USA; 4 Advanced Gastroenterology, Inc., Thousand Oaks, CA USA; 5 Saint Mary Medical Center, Hobart, IN USA; 6 Internal Medicine Associates, Merrillville, IN USA; 7 Indiana Medical Research, Elkhart, IN USA; 8 Unity Surgical Hospital, Mishawaka, IN USA; 9 Heartburn Center/Rehabilitation Department, Hancock Regional Hospital, Greenfield, IN USA; 10 Livingston Hospital and Healthcare Services, Inc., Salem, KY USA; 11 The George Washington University Medical Faculty Associates, 1800 Town Center Drive, #218, Reston, VA 20190 USA

**Keywords:** GERD, TIF, Heartburn, Transoral fundoplication, Regurgitation, Atypical symptoms

## Abstract

**Background:**

Four randomized controlled trials have demonstrated the short-term efficacy and safety of transoral esophagogastric fundoplication (TF) performed with the EsophyX^®^ device in eliminating troublesome gastroesophageal reflux disease (GERD) symptoms in well-selected patient populations. The aim of this study was to assess the durability of these outcomes at 3 years post-procedure.

**Methods:**

The TF EsophyX versus Medical PPI Open Label trial was conducted in seven US sites. Between June and August 2012, we enrolled patients with small (<2 cm) or absent hiatal hernias who suffered from troublesome GERD symptoms while on PPI therapy for at least 6 months and had abnormal esophageal acid exposure (EAE). Randomization was to TF group (*n* = 40) or to PPI group (*n* = 23). Following evaluation at 6 months, all remaining PPI patients (*n* = 21) elected to undergo crossover to TF. Fifty-two patients were assessed at 3 years for (1) GERD symptom resolution using three GERD-specific quality of life questionnaires, (2) healing of esophagitis using endoscopy, (3) EAE using 48-h Bravo testing, and (4) discontinuation of PPI use. Two patients who underwent revisional procedures by year 3 were included in the final analysis.

**Results:**

At 3-year follow-up, elimination of troublesome regurgitation and all atypical symptoms was reported by 90 % (37/41) and 88 % (42/48) of patients, respectively. The mean Reflux Symptom Index score improved from 22.2 (9.2) on PPIs at screening to 4 (7.1) off PPIs 3 years post-TF, *p* < 0.0001. The mean total % time pH <4 improved from 10.5 (3.5) to 7.8 (5.7), *p* = 0.0283. Esophagitis was healed in 86 % (19/22) of patients. At the end of study, 71 % (37/52) of patients had discontinued PPI therapy. All outcome measures remained stable between 1-, 2-, and 3-year follow-ups.

**Conclusion:**

This study demonstrates that TF can be used to achieve long-term control of chronic GERD symptoms, healing of esophagitis, and improvement in EAE.

Gastroesophageal reflux disease (GERD) is a chronic and often progressive condition that develops when the retrograde flow of gastric contents into the upper aerodigestive tract causes troublesome symptoms and related complications [1]. It is estimated that 20 % of the Western world’s population is afflicted by various degrees of GERD [2, 3]. Along with lifestyle modifications and over-the-counter remedies, proton-pump inhibitors (PPIs) are currently the mainstay of medical therapy and are particularly effective in controlling troublesome heartburn. However, recent studies have raised public concern relating to the potential development of osteoporosis [4], kidney disease [5], and dementia [6] with the continuous use of PPIs over the course of several years. Furthermore, and despite the unquestionable effectiveness of PPIs in the majority of cases, approximately 30–40 % of patients remain unsatisfied because of incomplete symptom control on optimized PPI regimens [7]. Patients and referring gastroenterologists are often reluctant to consider surgery as an option because of the occasional occurrence of debilitating post-fundoplication side effects [8, 9], which may account for the steady decline in the number of laparoscopic Nissen fundoplications (LNF) performed in the past two decades [10].

In this context, the transoral esophagogastric fundoplication (TF) performed with the EsophyX^®^ device (Redmond, Washington) has emerged as a viable endoscopic alternative to conventional medical and surgical treatments of chronic GERD. The TF procedure creates a full-thickness, partial esophagogastric fundoplication above the *Z*-line, with fastener fixations extending longitudinally up to 3.5 cm and circumferentially between 270° and 330° [11]. Published randomized controlled multicenter double-blind and open-label studies have demonstrated the superiority of the TF procedure as compared to high-dose PPI therapy and/or sham procedure in eliminating troublesome regurgitation and a range of classic and atypical manifestations of chronic GERD up to 12 months post-TF in subgroups of patients with hiatal hernias ≤2 cm [12–15]. Importantly, in more than 17,000 procedures performed to date, TF has maintained a solid safety record with virtual absence of associated new onset of dysphagia and gas bloat [12–15]. However, due to the lack of long-term follow-up data from the USA, the durability of the therapeutic effects following TF has remained in question.

The 3-year follow-up data from the TIF EsophyX versus Medical PPI Open Label (TEMPO) randomized trial (clinicaltrials.gov: NCT01647958), which are the subject of this report, offer the opportunity to explore the long-term effects and durability of TF. We analyze the variations in symptom control, healing of esophagitis, cessation of PPIs, and amounts of distal esophageal acid exposure (EAE) between baseline and at three time intervals: 1, 2, and 3 years post-procedure. To the best of our knowledge, the current study represents the longest reported follow-up for chronic GERD patients receiving the TF procedure in the USA.

## Methods

### Study design and oversight

Enrollment and follow-up of patients for the TEMPO randomized trial with a crossover arm was conducted at seven US centers from June 2012 through August 2015. The study design has been described previously [14, 15]. The trial was sponsored by the manufacturer of the EsophyX device which was used in performing the TF procedure. A protocol development team led by K. Trad designed the TEMPO study in collaboration with the sponsor. The trial protocol was approved by the institutional review board at each participating center. Data were collected by the investigators and coordinators at each site and entered into the secure electronic data capture system. As required by good clinical practices and applicable regulations, the sponsor of the trial was involved in source verification of the data (i.e., the comparison of reported trial data with information from primary health records of trial subjects). The first author wrote the initial draft of the manuscript, and revisions were made by all the authors. The study investigators vouch for the accuracy and completeness of the data and of all analyses.

### Patients

Patients 18 years of age or older who were experiencing daily troublesome regurgitation or extra-esophageal manifestations of GERD while on daily PPI therapy and who had abnormal EAE off PPI therapy (defined as pH <4 for more than 5.3 % of total recorded time using 48-h Bravo pH testing) were deemed eligible for the study [14]. Excluded were patients those who presented with a hiatal hernia larger than 2 cm in axial length or greatest transverse dimension, reflux esophagitis grade C or D (Los Angeles classification), Barrett’s esophagus >2 cm, esophageal ulcer or fixed esophageal strictures or narrowing. Additionally, patients with motility disorders and previous gastric or esophageal surgery were also excluded [14].

At the time of enrollment, patients were required to have a documented history of daily PPI use for at least 6 months and a confirmed diagnosis of GERD for at least 1 year. All participating patients provided written informed consent.

### Study procedures

Patients meeting eligibility criteria were randomly assigned, in a 2:1 ratio, to undergo TF (TF group) or to receive high-dose PPI therapy (PPI group). A computer-generated block sequence randomization of nine was used, and randomization was stratified according to study center [14, 15]. At 6-month follow-up assessment, all patients from the PPI group elected to undergo crossover to TF. We have previously reported data from the 6-month follow-up (comparing TF vs. high-dose PPI therapy) [14] and the 12-month follow-up (assessing durability of TF up to 12 months and assessing clinical outcomes 6 months post-TF in crossover patients) [15]. For the purpose of this study, we combined patients from the initial TF group and crossover group and assessed for durability of TF up to 3 years post-procedure. Clinical evaluation of all study patients was performed at the prespecified study intervals and according to the study protocol (Table 1).

**Table 1:** Evaluation protocol for patients enrolled in the study

Time interval	Symptomatic assessment		Esophagogastroduodenoscopy		48-h pH metry	
	On PPIs	Off PPIs	On PPIs	Off PPIs	On PPIs	Off PPIs
Screening (all patients)	X	X		X		X
6 months post-TF	X	X		X		X
6 months after high-dose PPIs and before crossover	X		X		X	
1-year follow-up	X^a^	X		X		X
2-year follow-up	X^a^	X		X		X
3-year follow-up	X^a^	X		X		X

All patients who were initially randomized to undergo high-dose PPI therapy underwent crossover to transoral fundoplication after completing their 6-month follow-up

*PPIs* proton-pump inhibitors, *TF* transoral fundoplication

^a^Minority of patients who were back on PPIs

The performance of the standard TF 2.0 procedure was mandated by the study protocol for all study patients who underwent TF [14]. All TF procedures were performed using the EsophyX device under general endotracheal anesthesia. The device is introduced transorally over a flexible endoscope and inserted into the stomach under constant endoscopic visualization. The endoscope is retroflexed to provide visualization of the gastroesophageal junction (GEJ). The helical retractor is engaged into the tissue just below the *Z*-line. The fundus of the stomach is then folded up and wrapped around the distal esophagus utilizing the tissue mold, the chassis, and the helix as an anchor. After locking all the tissue-manipulating elements, the invaginator is activated to allow the advancement of the GEJ below the diaphragm. Polypropylene “H” fasteners are then delivered through the thickness of the apposed stomach and esophageal walls. The maneuver is repeated at three additional positions to create a full-thickness, partial gastroesophageal fundoplication, with an average of 21 fasteners deployed at various locations. Intraoperative endoscopy is performed immediately before and after introducing the EsophyX device to assess the GEJ, confirm Hill grade, and assess the size of hiatal hernia, if present.

Patients were followed up for a maximum of 3 years post-TF. Following the last study visit conducted in September 2015, data were monitored for accuracy against source documents.

### Outcomes and effectiveness assessment

The primary outcome measure of the TEMPO trial was elimination of daily troublesome regurgitation and atypical symptoms, as defined by the Montreal consensus [1]. The Reflux Disease Questionnaire (RDQ) was used to assess frequency and severity of regurgitation; the Reflux Symptom Index (RSI) questionnaire was used to assess atypical GERD symptoms. RDQ is a 12-item questionnaire that was designed to assess the frequency and severity of heartburn (four items measuring the frequency and severity of pain and burning behind the breastbone), regurgitation (four items measuring the frequency and severity of acid taste in the mouth and movement of the material upward from the stomach), and dyspeptic complaints (four items measuring the frequency and severity of pain or burning in the upper stomach) [16]. Response options range from 0 (not present) to 5 (daily) for frequency and 0 (not present) to 5 (severe) for severity. Each patient’s score is calculated as the mean of item responses with higher scores indicating more frequent or severe symptoms. Troublesome symptoms are defined as mild symptoms, occurring 2 or more days a week, or moderate to severe symptoms, occurring more than 1 day a week [1]. The elimination of troublesome regurgitation was evaluated with the RDQ. A frequency score of three or more and severity score of two or more for the regurgitation questions were required to meet the Montreal consensus criteria for troublesome regurgitation [13]. RSI is a 9-item validated questionnaire used to measure atypical GERD symptoms such as hoarseness, throat clearing, excess throat mucus, dysphagia, and cough [17]. The scale for each individual item ranges from 0 (no problem) to 5 (severe problem), with a maximum total score of 45 and a normality threshold of ≤13.

Primarily, we assessed symptom control after the TF procedure with these questionnaires while patients were off PPIs at 1-, 2-, and 3-year intervals. Secondarily, in order to include data on patients who had resumed PPIs after TF and whose questionnaires were completed only while on PPIs, we also report symptom control regardless of PPI use (off or on PPI therapy).

Secondary outcomes included elimination of heartburn, healing of reflux esophagitis, cessation of PPI use, improvement in distal EAE, and patient satisfaction. Elimination of troublesome heartburn was assessed using the GERD health-related quality of life questionnaire (GERD-HRQL). GERD-HRQL is designed and validated to evaluate typical GERD symptoms by measuring ten items (six related to heartburn, two to dysphagia, one to bloating, and one to the impact of medications on daily life) on the visual analog scale ranging from 0 (no symptoms) to 5 (worst symptoms) [18]. A higher total GERD-HRQL score (range from 0 to 50) indicates more severe GERD [19]. We used endoscopy to assess and grade reflux esophagitis, if present. Complete cessation of PPI use was documented. Levels of distal EAE were assessed with 48-h Bravo pH monitoring, using percent total recorded time pH <4 as our main outcome measure, and considering 5.3 % as the threshold for normality. Patient satisfaction with their current health condition was reported as part of the GERD-HRQL, with three possible answers: satisfied, neutral, or dissatisfied.

### Statistical methods

The primary analysis in this report focuses on the stability of elimination of troublesome GERD symptoms up to 3 years following the TF procedure. The comparison within patients was made with the use of the repeated-measures analysis of variance (ANOVA) statistical test followed by post hoc Tukey–Kramer honestly significant difference multiple comparison procedure to identify which means differed from each other. In general, means and standard deviations (SD) are reported; the study follow-up intervals were reported as medians (ranges). Counts and proportions for the categorical data were compared with the use of McNemer’s test. The two patients who underwent revisional procedure (TF failure) were included in the analyses and were assigned the worst outcomes observed during the study from the timing of revisional procedure going forward. All analyses were performed using JMP 11.0 statistical software. *p* value <0.05 was considered significant.

## Results

**Fig. 1:**
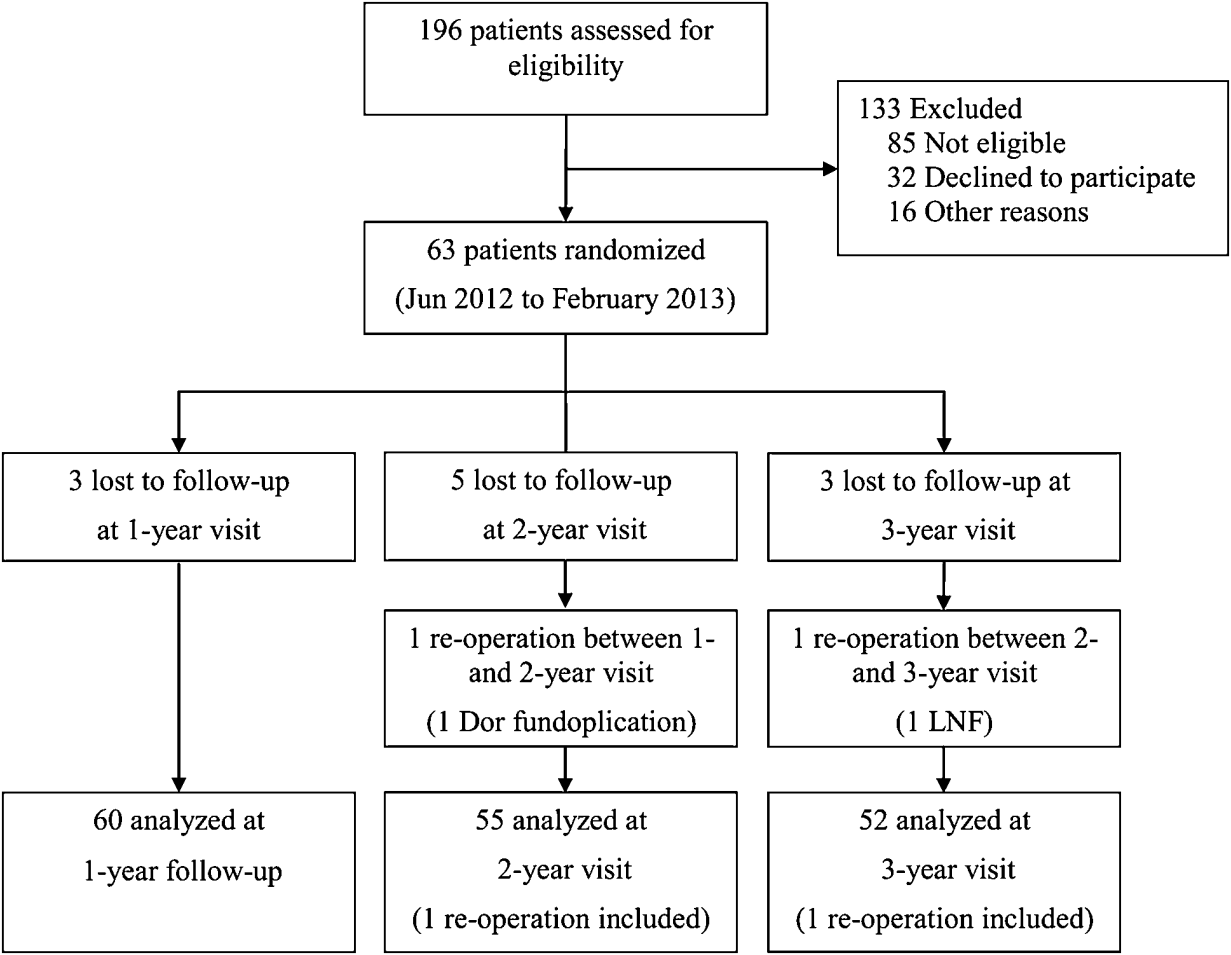
Study flowchart of treated and analyzed patients. Of the 85 patients not meeting eligibility criteria, 48 % (38/85) had normal pH test, 36 % (31/85) had hiatal hernia >2 cm in axial length or greatest transverse dimension, 13 % had Hill grade > II, 2 % had reflux esophagitis > grade B (Los Angeles classification), 2 % had body mass index >35 kg/m^2^, and 1 % (1/85) had Barrett’s esophagus >2 cm

Between June and August 2012, 63 chronic GERD patients were randomized (Fig. 1). Of these 63 patients, 60 (95 %) were available for analysis at 1 year; 55 (87 %) completed the 2-year assessment; and 52 (83 %) were available for the 3-year data collection. Before randomization, the average duration of GERD symptoms was 11.2 (9.8) years and the average PPI therapy duration was 8.6 (6.5) years (Table 2). Median time (range) post-TF was 11.2 (4.7–14.5), 22.8 (16.3–25.2), and 34.0 (27.0–37.0) months for the 1-, 2-, and 3-year follow-up intervals, respectively.

**Table 2:** Baseline characteristics of study patients

Characteristics	Study cohort (*n* = 60)
Female, *n* (%)	33 (55)
Age, years, mean (SD)	51.5 (10.3)
<50, *n* (%)	24 (40)
50–65, *n* (%)	31 (52)
>65, *n* (%)	5 (8)
Body mass index (kg/m^2^), mean (SD)	28.5 (3.7)
Gastroesophageal reflux disease (GERD) symptom duration (years), mean (SD)	11.2 (9.8)
Proton-pump inhibitor (PPI) therapy duration (years), mean (SD)	8.6 (6.5)
Barrett’s esophagus, <2 cm, *n* (%)	1 (2)
Esophagitis (Los Angeles grade), *n* (%)	33 (55)
A, *n* (%)	5 (5)
B, *n* (%)	28 (95)
Hill grade, *n* (%)	55 (92)
I, *n* (%)	7 (13)
II, *n* (%)	48 (87)
Hiatal hernia, *n* (%)	52 (87)
Axial length ≤1 cm, *n* (%)	17 (33)
Axial length >1 and ≤2 cm, *n* (%)	35 (67)
Greatest transverse dimension ≤1 cm, *n* (%)	17 (33)
Greatest transverse dimension >1 and ≤2 cm, *n* (%)	35 (67)
Patients on single dose of PPI at entry, *n* (%)^a^	43 (72)
Patients on omeprazole at entry, *n* (%)	26 (43)
Patients on esomeprazole at entry, *n* (%)	17 (28)
Patients on lansoprazole at entry, *n* (%)	6 (10)
Patients on pantoprazole at entry, *n* (%)	7 (12)
Patients on dexlansoprazole at entry, *n* (%)	4 (7)

^a^Of 12 patients in the TF group who were taking double-dose PPIs at entry, 6 (50 %) patients were on omeprazole; 3 (25 %) on pantoprazole; 2 (17 %) on lansoprazole; and 1 (8 %) on esomeprazole. In the PPI group, of five patients who were taking double-dose PPIs, 3 (60 %) patients were on esomeprazole and 2 (40 %) were on omeprazole

### Safety and procedure data

As previously reported, there were no serious adverse events (SAE) such as bleeding, abdominal or thoracic infections, or any other complication associated with the TF procedure [14, 15]. In this study cohort, a mean of 21 (4) fasteners were used to create valves with a mean length of 2.8 (0.5) cm and a circumference of 290 (20)° as assessed by the immediate post-procedure endoscopy. Ninety-five percent (57/60) of patients were released from the hospital within 24 h post-procedure. All 52 hiatal hernias were reduced with Hill grade II (*n* = 49) converted to Hill grade I in all patients. The rate of revisional surgery in this study was 3 % (2/60, Fig. 1) up to 3-year follow-up.

### Primary outcomes

**Fig. 2:**
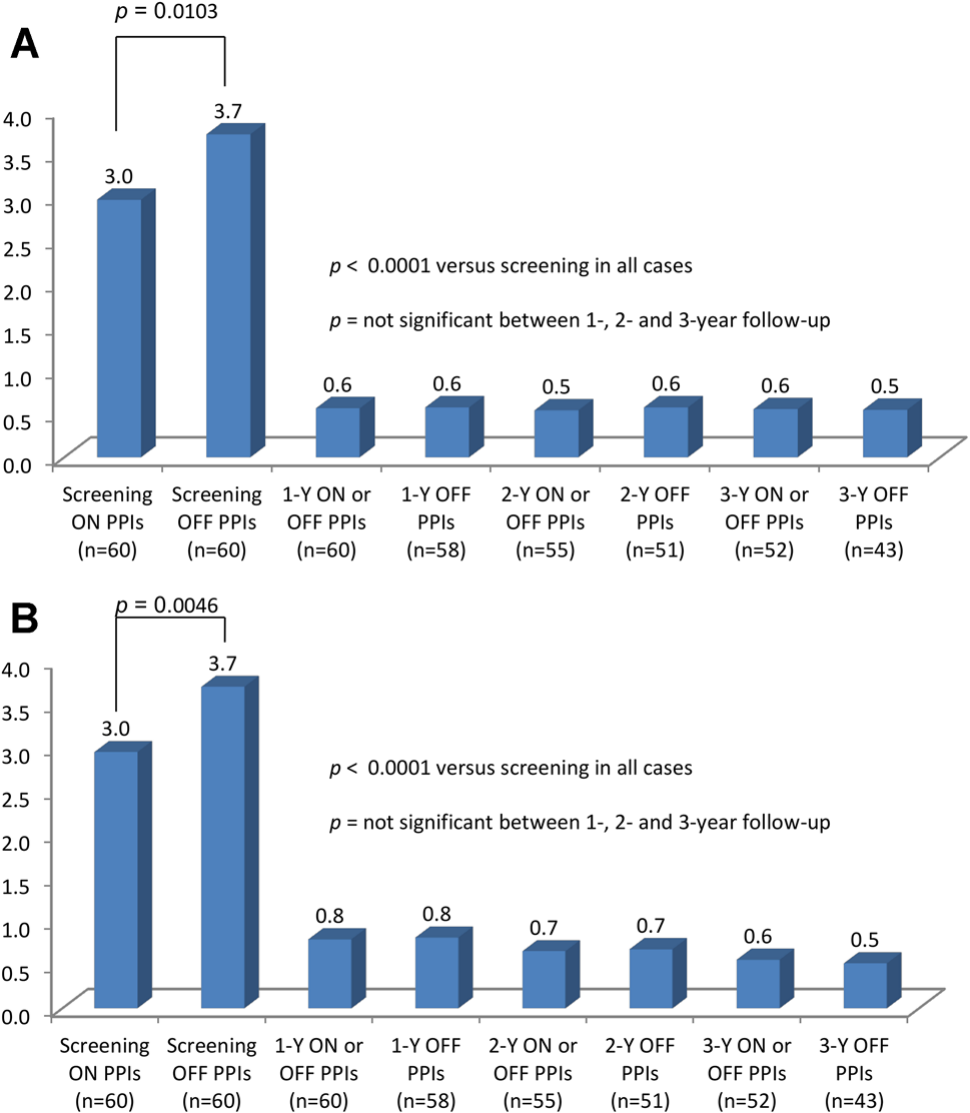
**A** Total regurgitation scores as evaluated by the Reflux Disease Questionnaire before and after transoral fundoplication in patients completing the study follow-up visits. **B** Reflux Disease Questionnaire total score before and after transoral fundoplication in patients completing the study follow-up visits

Elimination of troublesome regurgitation, as evaluated by the RDQ, was observed in 90 % (37/41) of patients at the 3-year assessment. Similar findings were observed at 2- (90 %, 41/44) and 1-year follow-up (88 %, 42/48). Elimination of troublesome regurgitation was further supported by improvement in the total regurgitation scores from 3.0 on PPIs at screening to 0.5 off PPIs 3 years post-procedure, *p* < 0.0001 (Fig. 2A). Improvement in the total RDQ score observed at 1-year follow-up remained stable between 2- and 3-year follow-ups (Fig. 2B).

**Fig. 3:**
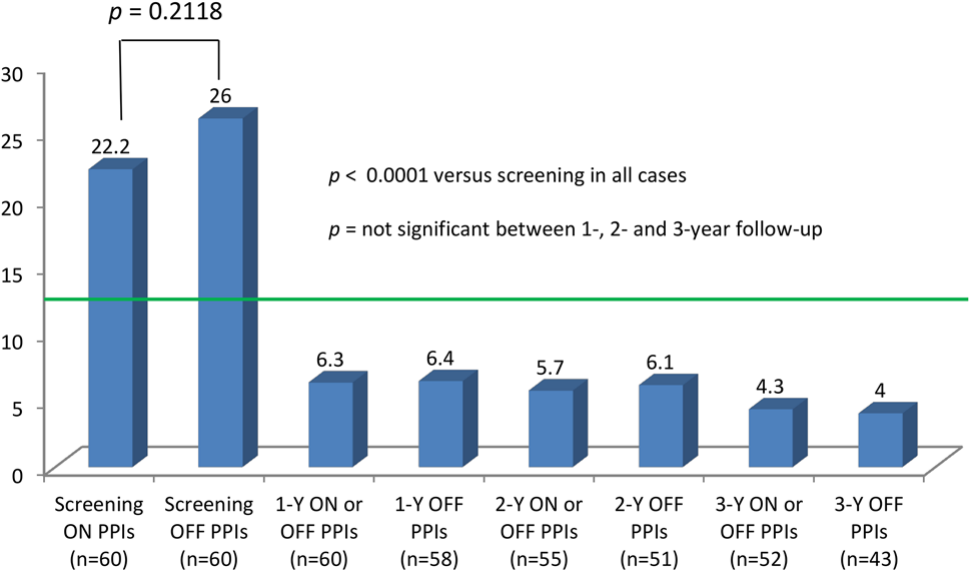
Reflux Symptom Index (RSI) scores in patients completing the study visits. *Green line* represents the normality threshold of 13 for the total RSI score

Elimination of all troublesome atypical symptoms (RSI score ≤13) was observed in 82 % (45/55) at 1-, 84 % (43/51) at 2-, and 88 % (42/48) at 3-year follow-up. Total RSI score improved from 22.2 (9.2) on PPIs at screening to 4 (7.1) off PPIs at 3-year follow-up, *p* < 0.0001 (Fig. 3). As was observed with total RDQ scores (Fig. 2B), total RSI scores showed no statistical difference in the means between 1-, 2-, and 3-year follow-ups, regardless of PPI use at the time the questionnaire was collected (off PPIs or on/off PPIs) (Fig. 3). Means of total symptom scores, as evaluated by the RSI questionnaires at the study intervals, are shown in Table 3. Global elimination of regurgitation and all atypical symptoms off PPIs was achieved in 83 % (48/58) of patients at 1, 82 % (42/51) at 2, and 83 % (34/41) at 3 years post-TF.

**Table 3:** Mean symptom scores at screening (on and off PPIs), 1, 2, and 3 years after transoral fundoplication (TF) off PPIs in study patients

Parameters	Screening (on PPIs)	Screening (off PPIs)	1 year after TF (off PPIs)	2 years after TF (off PPIs)	3 years after TF (off PPIs)	*p* value (3 years off PPIs vs. screening on or off PPIs)
Hoarseness	1.9 (1.6)	2.5 (1.5)	0.6 (1.2)	0.6 (1.2)	0.4 (0.9)	<0.0001
Throat clearing	2.9 (1.3)	3.3 (1.2)	0.9 (1.3)	1.1 (1.3)	0.6 (1.2)	<0.0001
Excess throat mucus or post-nasal drip	3.0 (1.4)	3.3 (1.2)	1.1 (1.3)	0.7 (1.2)	0.7 (1.3)	<0.0001
Difficulty swallowing foods, liquids, or pills	2.0 (1.4)	2.3 (1.5)	0.5 (0.9)	0.3 (0.8)	0.2 (0.6)	<0.0001
Coughing after eating or after lying down	2.4 (1.6)	2.9 (1.5)	0.6 (1.1)	0.7 (1.3)	0.5 (1.1)	<0.0001
Breathing difficulties or choking episodes	1.7 (1.6)	2.0 (1.5)	0.4 (0.9)	0.3 (0.6)	0.1 (0.4)	<0.0001
Troublesome or annoying cough	2.2 (1.5)	2.8 (1.6)	0.7 (1.3)	0.5 (1.1)	0.3 (0.9)	<0.0001
Sensation of something sticking or a lump in the throat (globus)	2.7 (1.4)	2.9 (1.4)	0.7 (1.2)	0.7 (1.2)	0.5 (1.0)	<0.0001
Heartburn, chest pain, indigestion or stomach acid coming up	3.3 (1.4)	4.2 (1.0)^a^	1.1 (1.5)	1.2 (1.7)	0.7 (1.4)	<0.0001

Values represent means (SDs)

^a^ *p* values between 1-, 2-, and 3-year follow-ups >0.05 in all cases except (*p* = 0.0038)

### Secondary outcomes

**Fig. 4:**
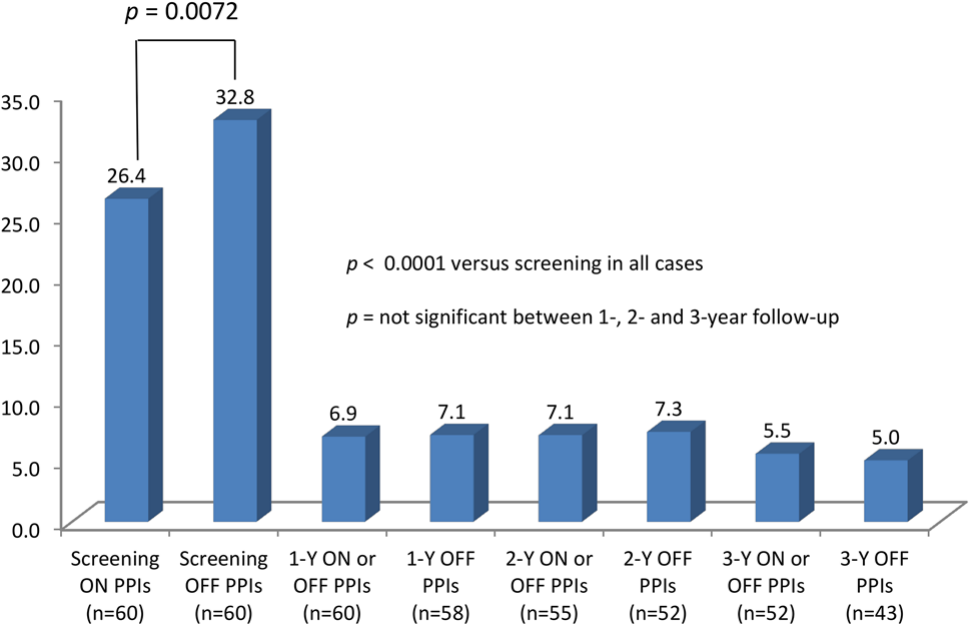
Gastroesophageal reflux disease health-related quality of life (GERD-HRQL) scores through the duration of study

GERD-HRQL improved from 26.4 (9.4) on PPIs at screening to 5.0 (9.2) off PPIs at 3-year follow-up, *p* < 0.0001. There was no statistical difference between the total GERD-HRQL score at 1-, 2-, and 3-year follow-ups (Fig. 4).

Of patients available for 1-, 2-, and 3-year follow-ups, 98 % (59/60) underwent endoscopic evaluation at 1-year, 91 % (50/55) at 2-year, and 79 % (41/52) at 3-year follow-up. Esophagitis was diagnosed in 55 % (33/60) of patients at pre-TF screening, in 5 % (3/59) at 1-year, in 10 % (5/50) at 2-year, and in 12 % (5/41) of patients at 3-year follow-up. Of 33 patients with esophagitis at screening, esophagitis healed in 94 % (31/33) with one patient presenting new onset grade A esophagitis at 1 year. At 2-year follow-up, esophagitis healed in 93 % (26/28) of patients; three patients presented with new onset of esophagitis compared to screening (two grade A and one grade B). At 3-year follow-up, esophagitis healed in 86 % (19/22); two patients who had esophagitis at 2 years were noted to have persistent esophagitis.

Seventy-eight percent of patients (47/60) were completely off PPI therapy at 1 year, 76 % (42/55) at 2 years, and 71 % of patients (37/52) had maintained their total discontinuation of PPI therapy at 3 years post-TF. The proportion of patients resuming PPI therapy did not change significantly from 22 % at 1 year to 24 % at 2 years, and to 29 % at 3 years post-TF (*p* > 0.05).

All esophageal pH parameters, with the exception of duration of longest reflux, improved significantly at 1 year post-TF as compared to screening and then remained stable through the duration of the study (Table 4). The improvement observed in duration of the longest reflux episode was statistically significant at 2- and 3-year follow-ups compared to screening. Rates of pH normalization were similar at 1 (41 %, 24/59), 2 (37 %, 18/49), and 3 years (40 %, 16/40) post-TF.

**Table 4:** 48-h pH parameters through the phases of the study

Parameters	Screening (*n* = 60)	1 year (*n* = 59)	2 years (*n* = 49)	3 years (*n* = 40)	*p* values (all intervals vs. screening)	*p* values (between 1-, 2-, and 3-year follow-ups)
Number of refluxes	169.8 (80.0)	117.1 (61.8)	106.6 (50.8)	105.1 (72.8)	≤0.0002	≥0.8201
Number of long refluxes (>5 min)	12.5 (6.2)	10.2 (7.2)	10.3 (8.1)	8.9 (7.4)	<0.0001	≥0.9763
Duration of longest reflux (min)	29.4 (15.0)	24.2 (14.8)	20.2 (23.4)	18.6 (19.1)	*	≥0.4298
Fraction time pH <4 (%)	10.5 (3.5)	7.6 (4.6)	7.7 (5.1)	7.8 (5.7)	≤0.0283	≥0.9944
DeMeester score	36.0 (12.2)	26.5 (15.2)	26.3 (16.3)	26.9 (18.2)	≤0.0173	≥0.9981

The values represent the total means (SD) based on 48-h pH testing

* *p* values: 3 years versus screening = 0.0171, 2 years versus screening = 0.0384, 1 year versus screening = 0.3794

Two percent of patients (1/60) at screening were satisfied with their current health condition as assessed by the GERD-HRQL questionnaire on PPIs. At 1-, 2-, and 3-year follow-ups, 76 % (44/58), 84 % (43/51), and 81 % (35/43) of patients were satisfied with present health condition off PPIs (*p* values <0.001, vs. screening on PPIs).

## Discussion

This 3-year report represents the longest follow-up on the TF procedure performed with the EsophyX^®^ device in the USA to date. In addition to quality of life assessments, it included analyses of variations over time in physiologic parameters, endoscopic evaluation of healing esophagitis, as well as rates of complete PPI discontinuation following the procedure. Perhaps most importantly, it addresses concerns about the durability of the outcomes after the TF procedure, which is relevant given the historical context of the poor long-term results of first-generation endoscopic plication devices such as the Endo-Cinch and the NDO Plicator [13]. Our study demonstrated that the TF procedure results in sustained, significant, and clinically meaningful elimination of troublesome GERD symptoms, healing of reflux esophagitis, and improvements in all pH parameters. The authors of this study believe that the quality of the data offers reassurance as to the prolonged benefits of TF up to 3 years post-procedure.

In addition to durable elimination of troublesome regurgitation and atypical symptoms, our primary outcome measure, this study provides evidence that the TF procedure is effective in the sustained elimination of troublesome heartburn, as evaluated by the GERD-HRQL (Fig. 4). Furthermore, prolonged healing of reflux esophagitis (86 % at 3 years) in this study and significant improvements in all pH parameters recorded over 48-h (Table 4) provide additional proof of the durability of the TF procedure. A disconnect (by a factor of 2:1) between the high rates of elimination of troublesome symptoms and the normalization rates of EAE achieved in this study may be viewed as problematic by some. It is important to note that several studies evaluating TF [15, 19], PPI therapy [20], and traditional laparoscopic fundoplication [21, 22] have demonstrated poor correlation between post-treatment pH parameters and symptom control as evaluated with various disease-specific symptom scores [13].

While elimination of troublesome symptoms and healing of reflux esophagitis are well-established and clinically relevant goals of GERD treatment [1], our studies [14, 15] and a report by Hunter et al. [13] suggest that symptom control may not require pH normalization. In fact, PPI therapy may be less effective in controlling abnormal EAE in certain patients than previously thought [15]. The rate of pH normalization off PPIs noted in our study 3 years post-TF (40 %) is comparable to rates of pH normalization reported in patients on double-dose PPI therapy (approximately 50 %) [20, 23]. These observations raise two important questions for future research. First, how much of pH improvement is necessary to achieve adequate symptom control and acceptable patient satisfaction? Second, does pH normalization have complementary effects on overall control of GERD? Uncontrolled GERD symptoms are a major reason for patients to visit their physicians and to utilize healthcare resources. We believe that the GERD therapies that are safe and able to provide control of GERD symptoms and prevent complications may reduce total medical and societal costs associated with the treatment of symptomatic GERD by reducing the frequency of outpatient visits and improving work productivity.

The published literature suggests that achievement of higher normalization rates of EAE after traditional anti-reflux surgery may be attributed to the creation of a supra-competent valve in such procedures; this may contribute to troublesome and sometimes debilitating dysphagia and bloating [13]. This is not the case in patients who receive the TF procedure, as de novo occurrence of post-procedure dysphagia and bloating is virtually nonexistent [14, 15, 24].

The results of the 6- and 12-month follow-ups of the TEMPO trial comparing TF with high-dose PPIs had established the superiority of TF in controlling regurgitation, heartburn, and atypical symptoms in a subgroup of chronic GERD patients with small (<2 cm) or absent hiatal hernias and with incomplete responses to PPIs [14, 15]. Similar results were reported in two other double-blinded randomized trials, one of which compared TF and placebo pills versus sham procedure and high-dose PPIs [13] and another comparing TF versus sham procedure in a European population [12].

The cohort of patients considered for our report consisted of the totality of patients enrolled in the TEMPO trial, merging the group originally randomized to TF with the initial high-dose PPI control group (of which all patients elected to undergo crossover TF). Both groups were statistically similar in every respect, allowing for this design [15].

All TF procedures in this study were completed without perioperative or long-term SAE reported, underscoring the safety of the procedure. There have been more than 17,000 procedures performed worldwide to date, without mortality and with an estimated SAE rate of 3.4 % in the published literature [24]. In this study, there were two reoperations (3 %); these patients were considered as failures of TF and included as such in our statistical analysis. Both revisional operations were performed without difficulty, confirming previous reports on the safety and feasibility of laparoscopic anti-reflux surgery after TF [25]. Recently, a study discussing another more invasive anti-reflux procedure purported that the rate of reoperation following TF could range from 11.5 to 52.6 % [26], citing reports from Europe where earlier iterations of the device and technique had been used [27–30] in a suboptimally selected patient population which included large hiatal hernias and Hill grade III and IV. In contrast, in the TEMPO trial and other recent randomized controlled TF trials [12–15], all patients received a standardized TF 2.0 procedure with a rotational component, as described by Bell and Cadière [11]. Further, TEMPO participants deployed 21 fasteners on average, in accordance with previous reports indicating a direct correlation between the number of fasteners and the durability of favorable outcomes [31]. Lastly, the quality of our results can be attributed to the adherence to strict selection criteria, particularly by excluding patients with hiatal hernias larger than 2 cm in either axial height or greatest transverse dimension, patients with Hill grade III or IV ratings of the GEJ, and patients with more severe erosive esophagitis (Los Angeles grade C or D).

We report in this study complete discontinuation of PPI use in 71 % of patients 3 years after TF, without statistically significant change compared to PPI cessation rates at 1- and 2-year follow-ups in the same group of patients. Testoni et al. [32] had reported that the percentage of patients who either stopped or halved their PPI therapy at 3-year follow-up was unchanged at 6 years (84 %), which portends positively for maintaining even longer-term PPI cessation rates in the same range. Interestingly, in the same study, complete discontinuation of PPIs dropped from 61 % of patients at 6 months to 30 % at 6 years, with the sharpest drop observed between 6 and 12 months post-TF, indicating that resumption of PPIs was most common within the first year after the procedure. In our view, this underscores the importance of patient selection and good technique. Furthermore, various factors make PPI use after any anti-reflux procedure an unreliable measure of success or failure, including easy access to over-the-counter medications and patients’ tendencies to resume PPI use without objective documentation of GERD. In fact, PPIs may be viewed an acceptable adjunct to TF procedures in patients whose GERD symptoms were uncontrolled on high-dose PPIs preoperatively [14, 15].

Limitations of the current study include its open-label, crossover design that may introduce a potential bias. While a comparison at 3 years of the medical (PPI) group and the TF group might have offered valuable additional data, there are several reasons for using a crossover design. Following enrollment, patients in the PPI group were required to take the maximum standard dose of currently used PPI in an attempt to optimize control of their GERD symptoms [14]. Patients experiencing ongoing symptoms despite medical therapy are routinely stepped up to maximum dosage in an attempt to control troublesome symptoms. Furthermore, the recommended treatment for patients with predominant extraesophageal symptomatology (our study population) is aggressive acid reduction using PPIs twice daily short term over a duration of 3–4 months [33]. A 3-year comparison between two groups would have likely made enrollment of patients and perhaps IRB approval very difficult. Furthermore, we elected to utilize a crossover design rather than analyzing two parallel groups to eliminate any potential confounding factors that may influence clinical outcomes [14]. Primarily, the TEMPO study was designed to evaluate efficacy of TF versus high-dose PPI therapy at 6- and 12-month follow-ups and secondarily to assess a durability of TF in all patients who underwent the TF procedure. Therefore, for the long-term outcomes each patient served as his/her own control. While we recognize that 11 patients were lost to follow-up for the 3-year evaluation, we believe that an attrition rate of 17 % is acceptable for any 3-year follow-up study. Additionally, as commonly seen in studies evaluating GERD therapies, not all patients were willing to undergo objective evaluation at the study intervals (Table 4); however, the consistent results between 1-, 2-, and 3-year follow-ups from this study, combined with the evidence from double-blind randomized studies [12, 13] and from the long-term European study [32], further support the safety, efficacy, and durability of TF. We conclude that TF offers chronic GERD patients with incomplete symptom control on PPI therapy an effective therapeutic alternative with lasting effect.

A study designed to provide a direct randomized comparison of the traditional anti-reflux surgery and the TF would be very difficult to enroll. Traditional anti-reflux surgery is often reserved for the patients with the most severe GERD, including patients with large hiatal hernias. In such patients, a diaphragmatic crural closure is routinely performed. In contrast, the use of the EsophyX device to perform the endoscopic TF procedure is restricted to a well-selected subset of GERD patients with small (≤2 cm) or absent hiatal hernia. This is reflected in the selection criteria of the TEMPO trial. Such patients are not typically referred for a surgical fundoplication; these patients receive a dose escalation of PPIs and addition of H2 blockers for breakthrough symptoms. In the case–control study of patients undergoing TF, Nissen, or Toupet fundoplication, TF achieved similar dramatic symptom resolution, when compared to Nissen or Toupet fundoplication; a shorter operative times and lengths of stay were observed after TF [34]. The TEMPO trial offered a randomized comparison of the two options for a common clinical scenario which presents significant challenges; it was conducted to find out whether TF is a viable alternative to patients with incomplete symptom control on optimized PPI therapy and who are fearful of the potential side effects of fundoplication. Furthermore, the level of scientific proof of its efficacy and therapeutic gain surpasses anything currently available outside the area of traditional laparoscopic anti-reflux surgery [12]. Based on currently available evidence, the authors believe that the TF procedure performed with the EsophyX device should not be considered experimental and should be offered to well-selected chronic GERD patients.

## Conclusion

Our study demonstrated that the transoral esophagogastric fundoplication procedure performed with the EsophyX device provides sustained symptomatic relief, healing of reflux esophagitis, and prolonged improvement in all esophageal pH parameters at 3-year follow-up. Our results further confirm the safety, efficacy, and durability of TF in well-selected symptomatic GERD patients on chronic PPI therapy. We conclude that transoral fundoplication should be considered in the management of GERD patients with similar disease characteristics as presented in this study.
